# Extra-osseous osteochondroma-like soft tissue mass of the patello-femoral space

**DOI:** 10.1186/1471-2474-7-57

**Published:** 2006-07-15

**Authors:** Francesco Oliva, Alessandro Marconi, Stefano Fratoni, Nicola Maffulli

**Affiliations:** 1 Department of Orthopaedics and Traumatology, University of Rome "Tor Vergata" School of Medicine, Viale Oxford 81, Rome, Italy; 2 I.N.I. Hospital Orthopaedic Unit, Grottaferrata, Rome, Italy; 3 Department of Anatomy Pathology "S. Eugenio Hospital" of Rome, Italy; 4 Department of Trauma and Orthopaedic Surgery, Keele University School of Medicine, North Staffordshire Hospital, Thornburrow Drive, Hartshill, Stoke on Trent, Staffordshire, ST4 7QB, England

## Abstract

**Background:**

Extraskeletal cartilaginous tumors are uncommon. Osteochondromas usually arise from the metaphyseal region of the growing skeleton.

**Case presentation:**

A 53 year old man presented with a three years history of anterior knee pain and inability to flex his knee more than 90°. Clinical examination and imaging studies revealed a nodular calcific mass in the anterior portion of the knee, displacing the medial portion of the patellar tendon. Following excision, histopathology confirmed the diagnosis of extra-osseous osteochondroma-like soft tissue mass, with no recurrence 24 months after surgery.

**Conclusion:**

An integrated clinical-pathologic diagnosis helps to clarify the nature of extraskeletal cartilaginous tumors that can arise at unusual anatomic site. Complete local surgical excision is the management of choice.

## Background

Osteochondromas usually develop in relation to the periosteum, and occur around the growth plate of long bones, especially the knee [1]. The tumor usually stops to grow with closure of the growth plate [1]. Intra-articular osteochondromas are rare in older individuals [2]. In joints with a large capsular space, such as the patellofemoral joint, osteochondromas can remain intra-articular [3].

The behaviour of extraskeletal osteochondromas is poorly characterised [4]. Furthermore, some of these tumors continue to grow after skeletal maturity [5].

We report a patient with an extra-osseous osteochondroma-like soft tissue mass in the anterior portion of the knee joint. The tumor displaced the patellar tendon and affected knee motion.

## Case presentation

A 53 year old man presented with a three year history of left anterior knee pain. He was unable to flex his knee more than 90°. He did not report any trauma, and serology did not reveal any metabolic or rheumatic conditions.

**Figure 1:**
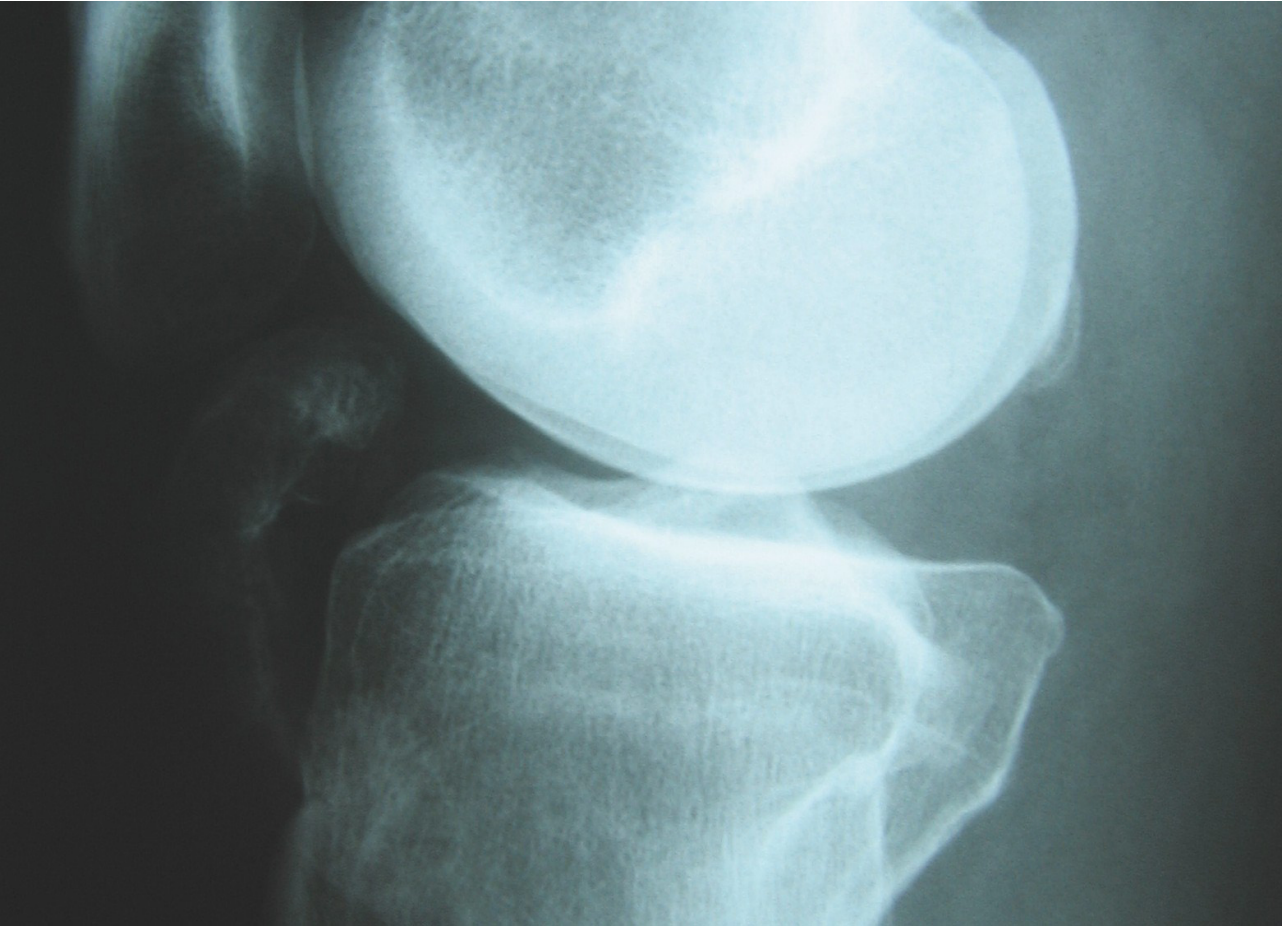
Lateral soft tissue radiographs at presentation.

**Figure 2:**
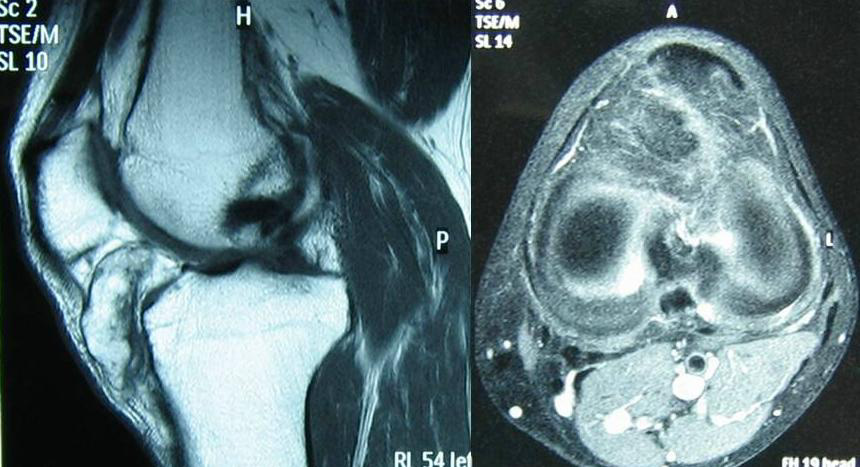
**A: **T1-weighted image: slight joint effusion. A mass located in the anterior portion of the space joint is evident. A pedicle is seen, but there was no continuity with the tibial plateau. The mass seems to contain some chondral components. **B: **T2-weighted image at the level of the tibial plateau shows intralesional bone formation (hypointense areas).

Clinical examination revealed a firm, nodular mass medial to the patellar tendon. The range of motion of the knee joint was limited to 90° of active flexion, with full extension. Neurovascular examination was normal. Plain radiographs showed a well delineated mass in the anterior portion of the knee. The femoral condyles and tibial plateau were normal (Fig. 1). The mass was well circumscribed, and was likely to consist of cancellous bone with small areas of radiolucency. MRI confirmed the presence of a pedicle, but there was no continuity with the tibial plateau. The mass was lying in the Hoffa body with the cap in close contact with the medial articular joint space, elevating and displacing anteriorly the medial one third of the patellar tendon (Fig. 2a–b).

Under regional anaesthesia with the patient supine in a bloodless field furnished by a tight tourniquet, a 5 cm longitudinal incision was made medially to the patellar tendon. The mass lied medially and behind the patellar tendon in Hoffa body. It was dissected and excised. The medial tibial plateau and the patellar tendon were not involved in the process. The capsule and the skin were sutured with subcuticular undyed 3/0 vicryl (Ethicon, Edinburgh, UK, EH11 4HE) and steristrips (3M, Loughborough, UK, LE11 1EP). A non adherent dressing, velband and crepe bandage were applied. Weight bearing was allowed in the immediate post-operative period.

**Figure 3:**
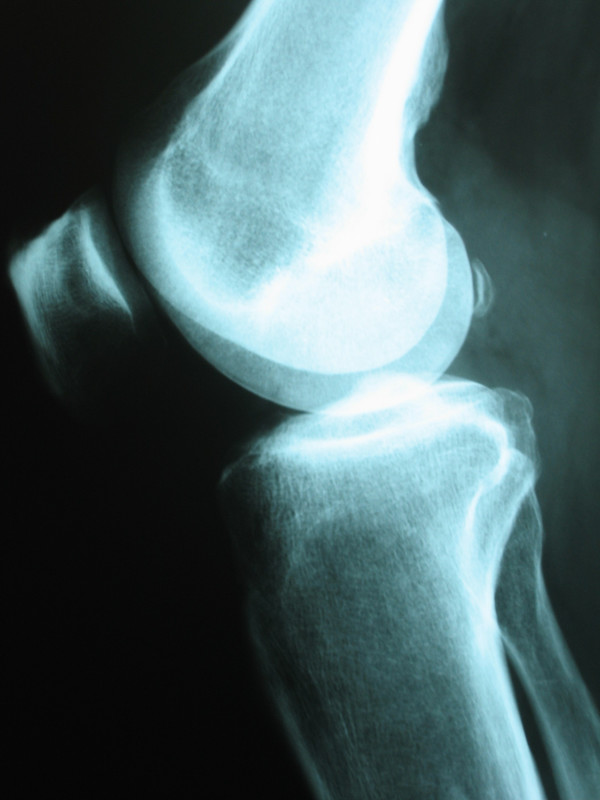
Lateral soft tissue radiograph 24 months after the operation. No recurrence is seen.

Post-operative recovery was uneventful. The patient returned to his activities of daily living, and regained full flexion two weeks after the procedure. When last reviewed 24 months after the excision, he was asymptomatic, with no clinical and radiographic signs of recurrence of the lesion (Fig. 3).

**Figure 4:**
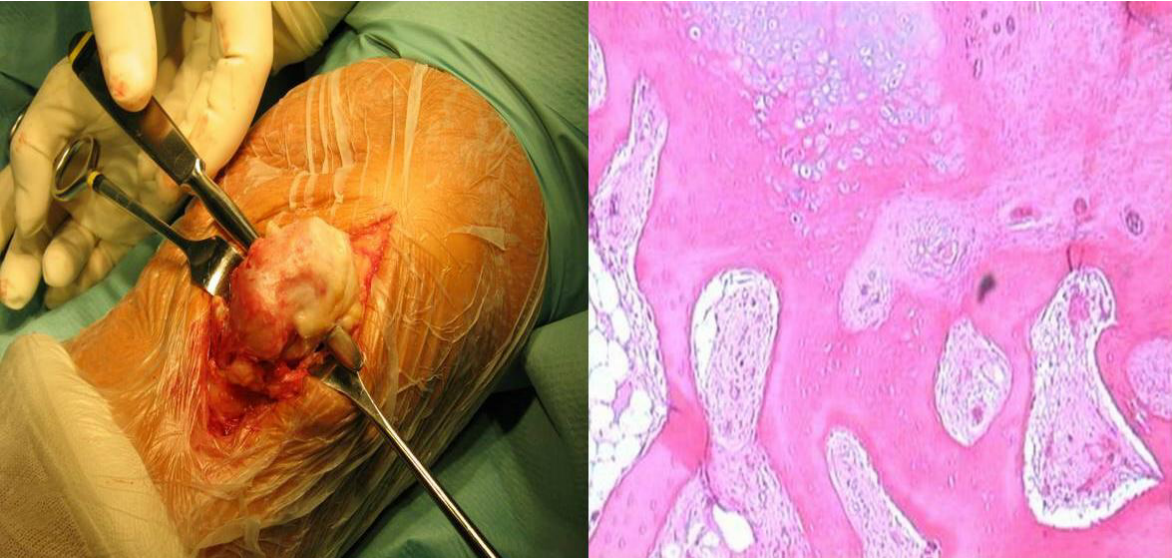
**A. **Macroscopic appearance of the tumor; **B. **Histopathology appearance (Haematoxylin-Eosin). A cap of mature hyaline cartilaginous tissue was covered by a fibrous membrane. The centre of the lesion is composed of mature bone trabeculae located beneath the cartilaginous cap, and containing bone marrow and amorphous calcified debris. Original magnification: ×10.

The firm mass showed a nodular appearance with a diameter of 7 cm with sharply demarcated edges (Fig. 4a). Microscopic examination showed a cap of mature hyaline cartilaginous tissue covered by a fibrous membrane. The centre of the lesion consisted of mature bone trabeculae located beneath the cartilaginous cap containing bone marrow and amorphous calcified debris. At the interface between mature bone and well-differentiated cartilaginous cap, there were foci of active endochondral ossification. There was no evidence of malignant features, and absence of mitotic activity (Fig. 4b).

## Conclusion

Osteochondromas usually arise from the metaphyseal region of the growing skeleton, with the medulla and cortex of the lesion being continuous with that of the parent bone. Continued growth after skeletal maturation can cause concern given the pre-malignant nature of osteochondromas. Most involve the knee region, although they may develop in any bone that forms by enchondral ossification. Usually growing away from joints, they are not articular lesions [1,2,5].

Extraskeletal osteochondromas are rare. Usually arising in the juxta-articular soft tissues without attachment to bone, these lesions may be large, and show the clinical and radiological features of a malignant process. Close to 40 extraskeletal osteochondromas not protruding into the joint cavity have been reported as para-articular, soft-tissue, capsular, intracapsular or intra-articular osteochondromas, ossification of the infrapatellar fat pad, and ossifying chondroma [1,5-13].

Only few intra-articular osteochondromas have involved the anterior and, more rarely, the posterior knee space joint. [1,3,14-19]. Bleshman and Levy reported an intra-articular osteochondroma of the hip with lateral displacement of the femoral head [2]. In our patient, plain radiographs showed a large well circumscribed, mineralised mass without abnormal calcifications within the adjacent tissues.

At MRI, there were no irregularities or thickening of the cartilaginous cap greater than 1 cm. Hence, there was no suggestion of malignancy [20]. The borders of the mass were well-defined, displacing the patellar tendon and the Hoffa fat pad without infiltration [20]. The size of the lesion and the small areas of chondroid tissue made synovial chondromatosis unlikely. In the differential diagnosis, malignant degeneration to chondrosarcoma had to be considered since the patient had reached skeletal maturity: by that time, proliferation of the cartilage should have ceased [1,5,6,20].

Intraoperatively, the tumor was completely intra-articular, well demarcated, with no evidence of continuity with bone. The gross appearance and histological examination demonstrated the features of an extra-osseous osteochondroma-like soft tissue mass with secondary bone formation in a fairly regular pattern through a process similar to normal enchondral growth [5]. The benign clinical course following removal, with no recurrence after 2 years, is in accordance with various reports [3,5,9,17].

Sarcomatous degeneration of osteochondral lesions such as synovial chondromatosis may be easier to identify by careful histological examination and correlation with the radiographic and operative findings, but clear histological features indicating the origin of an osteochondroma are not available [5]. Given the presence of the cartilage-free area of the pedicle, the osteochondroma may have originated at the posterior end plate of the tibia or femur, and grown towards the articular cavity of the knee. The pedicle could have then been torn so that the extra-osseous osteochondroma-like soft tissue mass became truly loose within the knee joint. Milgram and co-workers described such a pathogenesis for two intra-articular osteochondromas of the anterior knee joint cavity [3,17]. However, a soft-tissue origin has to be considered. Thus, in our patient the mass may have originated at the infrapatellar or intercondylar fat pad, growing through the anterior aspect of the joint. [16].

MRI is recommended for further characterisation of the nature and extent of an intra-articular osteochondroma. Operative removal is the procedure of choice when function is reduced and the nature of the tumor uncertain. In our patient, the absence of recurrence at follow-up after 24 months confirms the benign nature of the lesion.

In conclusion, an integrated clinical-pathologic diagnosis helps to clarify the nature of extraskeletal cartilaginous tumors that can arise at unusual anatomic site. Complete local surgical excision is the management of choice.

## Pre-publication history

The pre-publication history for this paper can be accessed here:
